# Experiences of persons in COVID-19 institutional quarantine in Uganda: a qualitative study

**DOI:** 10.1186/s12889-021-10519-z

**Published:** 2021-03-11

**Authors:** Rawlance Ndejjo, Gloria Naggayi, Ronald Tibiita, Richard Mugahi, Simon P. S. Kibira

**Affiliations:** 1 Department of Disease Control and Environmental Health, School of Public Health, College of Health Sciences, Makerere University, Kampala, Uganda; 2 Department of Epidemiology and Biostatistics, School of Public Health, College of Health Sciences, Makerere University, Kampala, Uganda; 3 Independent Public Health and Research Consultant, Kampala, Uganda; 4 Ministry of Health, Kampala, Uganda; 5 Department of Community Health and Behavioural Sciences, School of Public Health, College of Health Sciences, Makerere University, Kampala, Uganda

**Keywords:** Challenges, Coping, COVID-19, SARS-CoV-2, Experiences, Institutional, Quarantine

## Abstract

**Background:**

Quarantine has been adopted as a key public health measure to support the control of the Coronavirus disease (COVID-19) pandemic in many countries Uganda adopted institutional quarantine for individuals suspected of exposure to severe acute respiratory syndrome coronavirus 2 (SARS-CoV-2) to be placed in institutions like hotels and/or hostels of institutions for at least 14 days. This study explored experiences of individuals who underwent institutional quarantine in Uganda to inform measures to increase its effectiveness and reduce its associated negative impact.

**Methods:**

We conducted a qualitative description study using in-depth interviews with 20 purposively selected individuals who had spent time in institutional quarantine facilities. These were mainly phone-based interviews that were audio recorded and transcribed verbatim. Electronic data coding was conducted using Atlas.ti 7 software. Thematic content analysis was used to synthesize the findings with similar codes grouped to form sub-themes and ultimately study themes. The findings are presented thematically with typical participant quotes.

**Results:**

Study participants spent between 14 to 25 days in institutional quarantine. Four themes emerged describing the experiences of study participants during institutional quarantine, which determined whether participants’ experiences were positive or negative. These themes were: quarantine environment including facility related factors and compliance with COVID-19 measures; quarantine management factors of entity paying the costs, communication and days spent in quarantine; individual factors comprising attitude towards quarantine, fears during and post-quarantine and coping mechanisms; and linkage to other services such as health care and post-quarantine follow-up.

**Conclusion:**

The planning, management and implementation of the quarantine process is a key determinant of the experiences of individuals who undergo the measure. To improve the experience of quarantined individuals and reduce its associated negative impact, the pre-quarantine process should be managed to comply with standards, quarantined persons should be provided as much information as possible, their quarantine duration should kept short and costs of the process ought to be minimised. Furthermore, quarantine facilities should be assessed for suitability and monitored to comply with guidelines while avenues for access to healthcare for the quarantined need to be arranged and any potential stigma associated with quarantine thoroughly addressed.

**Supplementary Information:**

The online version contains supplementary material available at 10.1186/s12889-021-10519-z.

## Background

The Corona Virus Disease 2019 (COVID-19) epidemic that started in China continues to ravage the world and has caused over one million deaths, led to over 34 million cases by October 2020, and caused some of the greatest disruptions ever seen [1, 2]. In Africa, as of 6th October 2020, a total of just over 1.2 million cases and 26,475 deaths had been registered [3]. To deal with COVID-19, countries instituted several restrictions including lockdowns, strengthened disease surveillance and contact tracing, quarantined individuals suspected of exposure to severe acute respiratory syndrome coronavirus 2 (SARS-CoV-2) and treated and managed confirmed cases. Uganda registered its first case of COVID-19 on 21st March 2020 having already put in place restrictions one of which was to institutionally quarantine all persons travelling into the county from ‘high risk’ countries for COVID-19 transmission then with those from other countries recommended for self-quarantine [4]. This mechanism led to 2661 individuals being placed in institutions in several places across major towns including Kampala city, the country’s capital, and Entebbe, the airport city, mainly in hotels and hostels of institutions, until the international airport was eventually closed as part of the lockdown measures on 22nd March 2020.

Quarantine as a public health measure to control spread of diseases of epidemic potential is well documented dating back to the fourteenth century for control of plague [5, 6]. As opposed to isolation where individuals who develop symptoms are removed from the general public, in quarantine, individuals suspected of having had exposure to an infectious agent but are not showing symptoms are separated for observation for a period of time [6]. The measure has been used in the control of highly infectious diseases including the Spanish influenza pandemic, MERS and SARS, and its institution to control COVID-19 was thus not surprising [6–8]. Globally, quarantine is an unpleasant experience especially for people who undergo it. The change of environment, unpredictability of the infection status, physical isolation from loved ones and boredom are often associated with negative effects [9] and evoke emotions of fear, resentment, acceptance, curiosity and perplexity among others [6, 10].

During the lockdown instituted in Uganda, repatriation flights for citizens and residents stranded abroad were gradually allowed in the country from June 2020 until when the airport was officially opened on 1st October 2020. Returning residents were required to undergo mandatory institutional quarantine at their own cost, if they could not obtain the limited space in free government quarantine centres. In this study, we explored experiences of individuals who underwent institutional quarantine during this period in Uganda to inform measures aimed at increasing its effectiveness and reduce the associated negative impact.

## Methods

### Study area and population

This study was conducted in Uganda, a land locked country in the East African region with an estimated population of 41.8 million in 2020 [11]. The study population included individuals who had undergone institutional quarantine between July and August 2020. These individuals were selected from a mix of private and public institutions most of which were located within Kampala, the country’s capital, and the contiguous Wakiso district, the most populous district. The other quarantine sites were mostly along the land or water border points of entry run by government but airport travellers were quarantined in centres in Kampala and Entebbe.

### Study design and sampling

This was a qualitative descriptive study [12] that used in-depth interviews conducted with institutionally quarantined individuals. Participants were purposively sampled with maximum variation in mind considering institution of quarantine (private or public), sex, and age. All persons had exited the quarantined centres to ensure they provided a full experience. Participants were selected from a list of quarantined persons and their contacts obtained through the Ministry of Health. We called the selected participants to consent them to an appointment for interview.

### Data collection

Data were collected through individual interviews supported by a pretested interview guide (Additional file 1). In-depth interviews were suitable as the study aimed at describing individual experiences and behaviours [13]. The interview guide included open questions about individual experiences, challenges, opportunities, coping mechanisms, fears and perceptions of the quarantine period. Participants’ demographic characteristics were also captured including age, sex and marital status. Qualitative experienced research officers (2 females, 1 male) with master’s level education conducted phone interviews in English and/or *Luganda,* a local language, lasting about 25 min. One interview was conducted face to face. There was no relationship between the interviewers and the study participants. The interviewers used the guide but allowed the interview to flow naturally, actively listening while following up with appropriate probes [13]. All interviews were audio recorded with participant consent and later transcribed verbatim in English or simultaneously translated if they were in *Luganda* by the research team. We aimed and reached out to 30 participants, but five refused to participate while another five were not reachable by phone. Thus, 20 interviews were conducted; with saturation achieved by the 18th participant. Regular debriefs with the research assistants and the authors were helpful to determine if interviews still generated new information.

### Data management and analysis

All study transcripts were read several times by RN and GN, who together with SPSK had not been involved in data collection. We independently generated a codebook based on the themes laid out in the interview guide and following the study objectives. This helped to come up with an objective codebook devoid of biases. The codebook was then discussed and harmonized by the co-authors, and coding of the transcripts done following the semantic approach in Atlas.ti 7, allowing emerging codes to be added. At the end of the coding process, thematic content analysis was used to synthesise and group the codes into sub-themes, that followed partly the apriori set study themes [14] and some emerging ones. Typical quotes from the transcripts have been provided to support the study findings. Reporting for this study has been guided by the Consolidated Criteria for Reporting Qualitative Research guidelines [15] (Additional file 2).

### Ethical considerations

This study obtained ethical approval from the Higher Degrees Research and Ethics Committee of Makerere University School of Public Health (protocol 823) and was registered by the Uganda National Council for Science and Technology (HS 832ES). Study participants provided written informed consent and their privacy and confidentiality was ensured. Moreover, after audio recordings were removed from the phone, they were securely stored and later destroyed after verification of the study transcripts.

## Results

### Characteristics of study participants

Of the 20 study participants, 11 were female, 8 were aged between 29 to 40 years old, 16 were married and all except one had dependents. Fourteen study participants spent their quarantine in a private institution and only three were in quarantine for the stipulated 14 days with the rest spending over 14 days. All except one of our study participants were returning from outside the country, mostly from African (9), Middle Eastern (5) and European countries (4), and most had travelled for work-related reasons (17) or studies (3) (Table 1).

**Table 1:** Characteristics of study participants

Characteristics	Number
***Sex***	
Female	11
Male	9
***Age***	
29 to 40	8
41 to 50	6
51 to 65	6
***Marital status***	
Single	4
Married	16
***Occupation***	
Health professional	7
Business & sales	3
Student	3
Other professional	4
Other (fisherman, truck driver, retired)	3
***Had dependents***	
Yes	19
No	1
***Country/ place participant was returning from***	
African	9
European	4
Middle East	5
Other (India, within Uganda)	2
***Facility of quarantine***	
Public	6
Private	14
***Days spent in quarantine***	
14	3
15	9
Above 15	8

Eighteen of our study participants traveled back to Uganda through organized flights and were thus aware of the requirement to undergo quarantine on their arrival. The other two participants included a truck driver who was transporting goods from a neighbouring country and was stopped along the way, and a fisherman who was found in transit to another island within Uganda.

### Experiences in quarantine

**Fig. 1:**
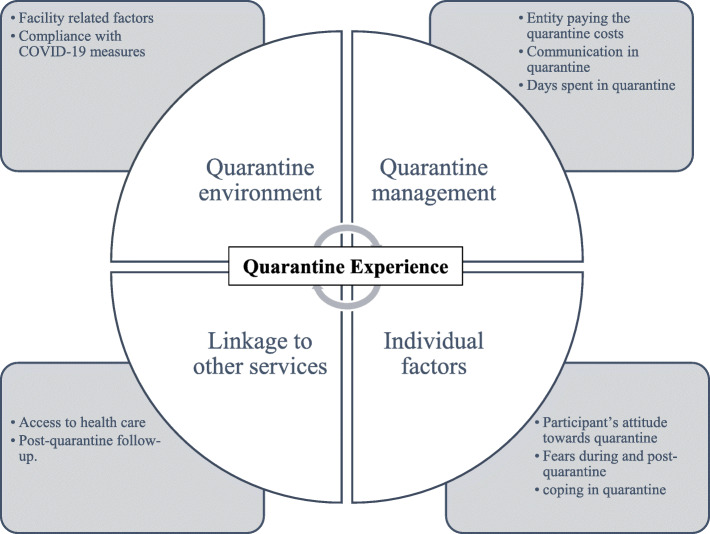
A summary of the study themes showing factors that influenced a respondent’s quarantine experience

Four themes described the experiences of study participants during institutional quarantine, which determined whether participants’ experiences were positive or negative. These themes were: quarantine environment, quarantine management, individual factors and linkage to other services as summarized in Fig. 1 and elaborated below supported with participant quotes.

#### Quarantine environment

The quarantine environment had two sub-themes of quarantine facility related factors and compliance with COVID-19 measures.

##### Facility related factors

Participants were in different quarantine facilities with variations in the facility environment including whether the facility had a compound or not, furnishings, the general hygiene and cleanliness of the facility, and the services and supplies provided including meals among others. Where participants felt that their needs were well catered for, they were positive about their experience.

*“My hotel facility was okay. I could buy food and my room had a balcony so I could rest without much interruption. My food was brought to the room and when I was tired of the room, I used the balcony or walked around the hotel. So, for me the ambiance was okay, it was a functional hotel, small but homely and not crowded which is what I wanted. So, my experience was good.”* (Female, 36 years, 15 days in private facility)

Other participants complained that the room size was small to allow exercising or the lack of a compound or balcony for them to connect with the outside environment. The lack of internet or pay television or having limited access to television channels also impacted on the quarantined persons’ experiences as some reported being bored while confined in their rooms.

*“My colleagues went to Kampala and had a good experience with excellent food and facilities. I regret choosing to stay in this place. At night there was a smell of sewage and I couldn’t open the windows and neither could I go to the veranda when I wanted to exercise. You don’t confine people in dark hotels for such a period of time. Hygiene was poor, they were not cleaning our rooms and they rarely changed our linen understandably. There was a smell of sewage which made me uncomfortable and extremely unhappy for the 14 days I was confined in this one facility.”* (Female, 58 years, 15 days in private facility)

The other prominent dissatisfaction reported was with meals where some quarantine facilities offered monotonous meals with no balanced diet/ variety, or provided unhealthy (fast) meals, and others provided no drinking water at meal times. To cope with these, some participants ordered for food and other necessities through online platforms or relied on their friends and relatives to deliver at the centres, where the restrictions allowed. Other participants also tried to engage the hotel establishment to change their meals which in some instances yielded results.

##### Compliance with COVID-19 measures

Another determinant of quarantined persons’ experiences was compliance with the COVID-19 prevention and control measures during the quarantine process by the authorities, the quarantine facility and quarantined individuals. Most participants felt that the COVID-19 prevention measures were not adhered to at the airport and during their transportation to the quarantine facility with many of them transported in vehicles without any physical distancing. This angered many especially because some had kept to these strict measures in the countries where they travelled from and during travel.

“O*n arriving at Entebbe Airport, they were just asking and telling us which hotels we were going to but then they put everyone in a single bus and those who arrived first had to wait almost for an hour to fill up with no physical distancing. Yes, a full board bus and I am like what? You know I have been maintaining physical distance and wearing my mask where I was for 3 months and now I am on this bus with people from all over the world and we were all being put on the bus with limited windows and aeration for almost two hours. They then start dropping off people going to their hotels as we wait for them to drop us.”* (Female, 41 years, 17 days in private facility)

At the quarantine facility, participants who were contented with how their facility complied with the available measures were more satisfied as they felt it complemented the purpose of the quarantine exercise and took it more seriously. They were also happier if their fellow participants complied with measures of staying within their rooms, wearing masks and social distancing. The army that was deployed at quarantine facilities also influenced compliance at individual level. On the other hand, where quarantine facilities had gaps in compliance with quarantine measures, participants were more disgruntled and doubted the motives of the quarantine measure which impacted their experience. Reported gaps among facilities were majorly around: cleaning especially in private institutions and hotel staff not following the recommended measures. Moreover, in public facilities, since hygiene facilities were shared, they were well cleaned and participants reported satisfaction with their state. However, there were complaints of other quarantined persons in some public places having access to drugs and alcohol which affected the experience of others.

*“Yes, it [quarantine experience] was good, the only worry we had about the government facilities, we thought they would put us like in a hall all together and you had to fight your own way of survival but we later found out that you are given your own room, the compound was big and you could physical distance. It was okay, the facilities were clean; they would clean them in the morning and in the evening.”* (Female, 40 years, 17 days in public facility)

There were also suggestions of other quarantined individuals being given preferential treatment compared to others including them seeing their family or being allowed to go for self-quarantine which caused some dissatisfaction among participants.

#### Quarantine management

The quarantine management factors were entity paying the costs, communication while in quarantine and days spent in quarantine.

##### Entity paying the costs

Quarantine costs including accommodation and meals were either paid by the individual themselves or their employer for those in private facilities or the government for those that opted for public facilities. The participants noted that the hotel costs were exorbitant, and they had been given limited choices of facilities to choose from. Where the quarantine costs were not being borne by the participant themselves, even when they acknowledged that the costs were high, they reported a more positive experience compared to their colleagues who paid the costs by themselves.

“*The quarantine costs were expensive but mine was not coming directly from my pocket but rather my employer. Generally speaking, compared to the rest of other places, this is expensive but the pain and burden of the costs rested with my employer*.” (Male, 29 years, 15 days in private facility)

Other participants noted that since all their basic necessities were covered, they never faced financial challenges.

*“We never had financial issues because we were given food, we had breakfast, lunch and dinner. We were given tea, soap, mosquito nets, blankets even sanitary pads. We thus did not have financial challenges because we had the essentials unless someone wanted to drink wine that’s when you had to dig into your pocket.”* (Female, 47 years, 17 days, public facility)

On the other hand, where participants paid the costs themselves, they were dissatisfied and others had to pay for other bills such as electricity on top of the full board accommodation costs. To cope, some of the participants reduced the number of meals they had in a day.

*“The hotel was expensive charging us $105 per night and the meals were charged separately and still expensively. We thus resorted to eating breakfast and an early dinner because we were not doing anything and so we didn’t have all the meals. But it was expensive and at the end of my 16 days, the cost was $1860 which is a lot of money that I am trying to claim from my employer but I don’t know about other people.”* (Female, 65 years, 16 days in private facility)

##### Communication

One of the key determinants of participants’ experiences in quarantine was related to the quality of communication they received. Most participants reported communication gaps regarding quarantine preparation, expected quarantine days, and COVID-19 testing and receiving results. The other gaps were in: extension of the quarantine period and expected dates of departure, information on COVID-19 preventive measures, and where they would obtain their confiscated passport. These communication gaps negatively impacted the experience of quarantined individuals.

*“They take off your samples expecting to get results … because they took our emails at the airport so I thought that maybe they would send results on email but what we got was a verbal communication. They were like ‘since none of you has been taken out of the facility by now, it means you are all okay’ (laughs) … This came after three days of asking for the results which had been promised in 24 hours. In fact, we did not get the first test result and only got the departure results from the hotel.”* (Female, 34 years, 16 days in private facility)

Many participants also reported not receiving any briefing about how to conduct themselves in quarantine and keep safe from COVID-19. Others said that they were simply handed leaflets with information to read which mode did not favour everyone while some obtained information through internet and the media. Participants who had sufficient information to guide their decision making and understand the quarantine process, and those who received regular updates regarding decisions that could affect them reported a more positive experience in quarantine. In fact, some participants reported receiving extra information through their employers who had prepared guidance for them in advance. Some of the participants had also undergone trainings on COVID-19 as health workers which supplemented the Ministry of Health information.

##### Days spent in quarantine

The Ministry of Health guidelines indicated that a minimum of 14 days in quarantine was expected of persons in institutional quarantine. However, among the study participants, only three spent 14 days in quarantine with the rest having their quarantine duration extended. This extension was attributed to the need to obtain COVID-19 test results for samples taken on the 14th day. Other extensions were based on finding a positive case within the quarantine facility as the guidelines stated a re-start of the quarantine period in such instances. Participants reported anxiety, stress and anger related to their quarantine days being extended and others protested the extension. Their experiences were further compounded by poor communication received from the authorities and their need to incur more costs especially where they were paying for themselves. Other participants had to start new engagements with their employers to cover the extra costs and others had to use their leave days as the extended period had not been catered for by their employer.

*“There was a delay of the results but the formal quarantine days were 14 so when we delayed we had an additional day and yet you have prepared your mind and already communicated to everyone that on the 14*^*th*^ *day you are going home but you don’t go and so it brought with it anxiety to the family and myself but I later got over it.”* (Male, 29 years, 15 days in private facility)

Some participants had invited their families to pick them but this was not possible due to the extended quarantine duration.

*“Of course I was not happy because I expected to leave on Sunday. I even called my son and he drove to Entebbe but when I realised that it was coming to 4pm and the results had not been delivered, I had to tell him to return to Kampala because of the curfew. So, I was not happy as I had already spent several days in quarantine in another country before coming back to Uganda.”* (Male, 58 years, 15 days in private facility)

#### Individual factors

Regarding individual factors, the attitude towards quarantine, fears during and post-quarantine and coping measures while in quarantine were important.

##### Attitude towards quarantine

Having known about the requirement to undergo quarantine as a public health measure prior to their travel, almost all participants reported preparing for it and were positive acknowledging that some had come from areas with high infection and were glad they were protecting the population and their own families. This attitude contributed to their acceptance of the situation and coping with the measure.

*“I did not feel bad or anything. Actually, I was glad that at least they are protecting Ugandans from this COVID-19 epidemic. Moreover, I would also want the same for my people to stay safe as we continue to bring back those stranded from outside the country and so the quarantine centers were a good idea.”* (Female, 31 years, 16 days in quarantine in public facility)

However, other participants had concerns about the procedure saying that they had already been tested from their departure countries and were negative and were not happy being put in the same facility with others who did not know their status.

*“I already knew that people coming back had to be quarantined but we left our destination after testing for COVID-19 and we were negative and so it is a challenge coming back here and you are told that those results are not being considered and we needed to be quarantined for 14 days in a place with other people who did not know their status but since it was a government decision, we had nothing to do.”* (Female, 40 years, 17 days in public facility)

The experience was however different for the participants who had not been prepared for the measure as they were taken into quarantine from attending to their activities such as the truck driver and the fisherman who later reported accepting the measure.

##### Fears during and post-quarantine

Almost all quarantined persons had fears of either having COVID-19 as some had not been tested beforehand or getting infected while in quarantine as they knew they were in a high-risk environment. Having initial negative test results prior to quarantine or early during quarantine comforted and helped some cope that they did not have the virus. Participants having been told that a positive case in their centre would lead to an extension of the quarantine period made them fearful while others were worried of the stigma that awaited them in their communities. The fears were compounded in some instances by some not having medical insurance cover or uncertainty of what the infection would mean to them and the continued admission of newer persons to their quarantine facilities.

*“My major worry was that they brought new people in our quarantine centre daily whose status we did not know, and we were never separated. Everyone had their own room but we were mixed. They could not group us like this batch has already spent 7 days here and should occupy this space or the other. We were sharing the same facilities and we worried that if anyone was infected amongst us, we would all have to redo the quarantine yet we had already done several days. It brought anxiety and people were fearing each other. We tried to do all things such as distancing and washing hands with soap and water.”* (Female, 40 years, 17 days in public facility)

One person reported fear in communicating with their family and friends saying they had been told that their communication regarding quarantine would be tapped by authorities. Study participants also strongly worried about being infected by the hotel staff who served them as many still had contact with the outside environment and did not properly observe the protective measures expected of them.

“*You do not control the people serving you, you do not know whether they are observing social distancing or washing their hands or wearing a face mask and they are serving all of you. You do not know whether they are staying in the hotel or coming from out, so it always brought the worry. Food is delivered to you but you have no idea how it was prepared and this is not in your control. You are in quarantine but there is a risk of you being exposed much as you are observing other measures well. Sometimes the person bringing food in the morning is different from that bringing the lunch, and dinner is also brought by a different person. At least they minimised cleaning the rooms and changing bed sheets which reduced chances of disease transmission.”* (Female, 52 years, 14 days in private facility)

On the other hand, a few participants reported no worries with some saying they were in a very small hotel with a few people and thus the risks were minimal, and others believed they had good immunity and did not have underlying conditions and so could fight off the virus if infected.

The post quarantine fears ranged from anticipated stigma or violence from the highly alert community. Some participants thus took steps to contact the local authorities about their situation or report to them with their discharge certificates soon after quarantine. Some participants were confident that their communities did not know they had travelled and others moved with their test certificate.

“*I felt a bit stigmatised especially in the community but within Kampala, my sister received me very well but the problem now is that I want to head to the village. I called the local area chairperson because in that area when they see someone coming from outside the country, the community rejects you. The Chairperson told me he was going to make announcements in the community and then when I go, the leaders will receive me and take me home as I have my certificate.*” (Female, 52 years, 14 days in private facility)

A few participants were fearful of contracting COVID-19 from the community as they were certain of their status having obtained their negative test results. This however also gave others confidence not to worry especially about infecting their family who would not stigmatise them. Due to being very self-conscious, some participants worried about being seen wearing a face mask around in public as they could be assumed to be carrying the virus and some resorted to telling those they met not to fear them as they were negative or simply keeping a low profile within their communities. Other participants had fears of how they would start all over again as some had lost their jobs in the countries where they were travelling from or had overspent during quarantine and finances were on top of their worries.

##### Coping in quarantine

Quarantined persons employed several measures to cope with their situation at different levels. Many of the participants started by preparing themselves psychologically before getting into the quarantine and accepting the situation as a mandatory government policy that they could do nothing about. Within the quarantine, many adopted a routine to keep active and pass time, others kept in touch with their friends and family through frequent phone calls but noted to sometimes be the ones comforting their families, or resorted to work or study which they said lessened the burden of the quarantine days. Other participants relied on their previous experiences as some had experienced quarantine elsewhere or had lived in conflict settings where they had learnt survival skills. Some participants noted viewing quarantine as an opportunity to progress with work or studies, reflect on one’s life, have adequate rest, write about their experiences and make friends and network.

“*Aaaah.,. you just have to adjust to the situation you are in and loosen up. You have to get up like in a boarding school, go to bathe and be ready for breakfast. After breakfast you do whatever you want with yourself, the social distance was there and you are not allowed to talk to people so much. We just adapted and found ways of living with it.”* (Female 31 years, 16 years in public facility)

The other reported coping mechanism were exercising within rooms or in the hotel compound, use of social media and the internet including attending online classes as some hotels had reliable internet, watching television or movies, and sleeping. Additionally, peer support was mentioned to have been key in supporting coping with many socialising among themselves and sharing challenges and experiences while supporting one another. To facilitate this, some quarantined persons created a WhatsApp group to stay in touch within quarantine. Others could sometimes meet and pray together. The Ministry of Health also sent through counsellors but only a few quarantined persons mentioned that this helped them cope.

*“During quarantine, we would gather around in the morning about 10 o’clock and share some scriptures and worship while observing social distance and everyone would put on a mask. We then repeated the routine in the evening.. We also created a WhatsApp group where we could encourage each one to be strong that we could get out of quarantine. The group is still active up to now, and people keep checking on each other asking ‘how are you? how is everyone? are you okay?’”* (Male, 35 years, 15 days in public facility)

#### Linkage to other services

Linkage of persons to health care while in quarantine and the post-quarantine follow-up also influenced the experience of quarantined persons.

##### Access to health care

The quarantined persons had other health needs and some requested for medications, monitoring of their health conditions such as diabetes and health checkups. Other times they had medical emergencies and required to be attended to. Where required services were provided, they were more satisfied and the reverse was true where the services were not provided.

*“Of course, on the second day of my arrival, they had to check on the heartbeat of the baby to feel whether the baby was doing well. The doctor came back to do a scan to confirm if the baby was still okay and check on those of us who were on medication to see if we needed refills and it was really all good. Personally, I am grateful.”* (Female, 31 years, 16 days in public facility)

*“I really needed medical care after an asthmatic attack as I had chest pain and I couldn’t even rotate in bed. When I requested for help, the health worker who comes here every morning to take our temperatures planned to take me to see a doctor but they refused him from doing so. The army officer, who I think is also a health worker, brought me some medication.* (Female, 52 years, 14 days in private facility)

##### Post-quarantine follow-up

Some participants were followed up afterwards by the Ministry of Health team to check on how they were doing or ask how their 14-day quarantine was or provide more quarantine related information. However, participants who received such calls reported a bad experience as some were angry about their quarantine experience and did not want to speak to the Ministry of Health officials. Moreover, these calls were sometimes automated, frequent and uncoordinated and some were a continuation of the calls they had received during quarantine which they felt was an unnecessary bother.

*“After quarantine, it is absolutely annoying to be called by the Ministry of Health to tell you that ‘hello friends, this is to tell you that you are highly suspected to be … because you travelled’ that is stigma for me. If I am seated with my friends and I get such calls, they might be like ooh … you know. This is after 2 months you are still getting those annoying and embarrassing calls. Till today, even when I am seated with my friends, family, or in the supermarket, I pick up the call and they say “hello friends, you know … ” I switch it off and for me that is stigma and it is terrible (laughs)”.* (Female, 58 years, 15 days in private facility)

Moreover, owing to the extended quarantine duration and having obtained their negative COVID-19 test results, many of the participants were reluctant to observe another 14-day self-quarantine period as was required by the Ministry of Health guidelines post institutional quarantine.

## Discussion

This study explored experiences of persons in COVID-19 institutional quarantine in Uganda and found four themes that influenced the experiences of quarantined persons. These themes were: quarantine environment, quarantine management, individual factors and linkage to other services. The study findings suggest that the planning, management and implementation of the quarantine process is a key determinant of the experiences of individuals who undergo the measure and where this is well considered, the negative experiences can be significantly minimised.

In our study, the stressors in quarantine were: non-conducive facilities with inadequate provisions and poor compliance with disease prevention and control measures, having to pay for quarantine costs, poor communication from authorities and extension of the quarantine period. In addition, a poor attitude towards quarantine, fears of infection and stigma, boredom and poor coping mechanisms, the lack of access to health care, and unnecessary post-quarantine follow ups were reported as stressful. These experiences are similar to those reported in recent rapid reviews that noted a longer quarantine duration, infection fears, frustration, boredom, inadequate supplies, inadequate information, financial loss, and stigma as key stressors in quarantine [10, 16]. Such experiences can lead to feelings of isolation, anger, frustration, confusion and traumatic stress symptoms negatively impacting quarantined individuals [8, 10, 16, 17] including in the long term [10, 18, 19] and their community. Quarantine though remains a key measure in the COVID-19 response with high potential to impact disease transmission and trends [20, 21]. Based on our research findings, in order to ensure a positive experience for quarantined persons and reduce the negative impact of quarantine, we suggest the following measures:

- *Manage the pre-quarantine process:* The pre-quarantine process in this study involved being received at the airport, screening and testing for COVID-19 and transporting persons to quarantine facilities. This process should be well managed and follow the recommended standard operating procedures such as appropriate screenings, sufficient physical distancing and wearing of face masks as it is the first encounter of participants with the process to which they need to have confidence.
- *Share as much information as possible:* Quarantined individuals need accurate and consistent information about the quarantine process, the duration of the quarantine including when such a period would need to be extended, and how they are expected to conduct themselves. Where testing is done, information should be clear on how they would obtain their results and relay them to reduce any anxieties associated with status uncertainty. Even when the quarantine period requires to be extended, there should be proper empathetic communication to the quarantined including information, education and communication materials in an understandable language and support to help them deal with anticipated effects. Sufficient information and support should also help the quarantined adopt a positive attitude towards the measure, deal with any fears they have and share coping mechanisms that can ensure a less stressful quarantine. A person’s knowledge about COVID-19 including its transmission and symptoms, and quarantine protocols increases their likelihood of compliance with quarantine [22]. A review of the psychological impact of quarantine in the US and UK found that people in quarantine with fear of infecting their loved ones coped easily when public health officials provided adequate information about the disease in question and the need for quarantine [23].
- *Keep quarantine duration short:* In planning for quarantine, the quarantine duration should be kept as short as possible to minimise the impact of long periods of isolation. This should be consistent with the science and how it evolves and protocols should factor this as a principle in determining quarantine stays. It is also important to avoid extending quarantine days except when absolutely necessary. Other potential workarounds could be considered such as prescribing self-quarantine or using a blend of time in institutional quarantine followed by another in self-quarantine.
- *Support quarantine costs / minimise costs*: Quarantine is a costly undertaking and where the costs are pushed on to the individual, they may lead to huge effects on their financial status from which they may not easily recover further impacting them psychologically. Where possible, governments should provide basic quarantine centres which meet standards that they can fund or offer a variety of low-cost facility options which participants can pay for without much strain. Loss of income and effect on work remains key concerns among quarantined individuals [10, 22].
- *Adequate preparation and monitoring of quarantine facilities:* Before centres are used for quarantine, they should be inspected to ensure they meet basic standards that can support quarantine. The centres should be trained on how best to support the quarantine process and adhere to required prevention and control measures. Further information should be provided on services for quarantined persons such as appropriate and healthy meals and supplies, and the quarantine dos and don’ts among others to support them in dealing with boredom.
- *Ensure access to health care:* While in quarantine, persons will continue to have other health needs that need to be broadly planned for including the procedures to be followed to obtain these services, provision of first aid facilities and any emergency needs. These should be considered in planning for institutional quarantine. Provisions for social and psychological support should also be incorporated to support individuals to cope better.
- *Address any potential for stigma:* The authorities should be upfront to deal with any potential stigma that may be associated with being in quarantine as this is likely to influence compliance and willingness to undertake the measure and overall experience of quarantined individuals similar to recent research findings [22].

### Study strengths and limitations

Our in-depth interviews were mainly conducted remotely over the phone due to the COVID-19 restrictions on social distancing and our study participants being from different areas of the country. This meant a shorter interview duration and a reduced opportunity to take note of participants’ nonverbal communication. Our study population pool was mainly of international travelers, who at the time were the most in quarantine facilities. These generally are of relatively higher social economic status than the average population. Our selected participants indeed were majorly educated, employed with international organisations and several were health professionals. These features may render their experiences a little different from those of different social characteristics. Moreover, our participants though could have been from anywhere, being quarantined in facilities within Kampala and Wakiso districts could have conferred on them a different experience compared to those in other quarantine centres along land or water border points. This notwithstanding, the influencing factors of the quarantine experience are likely to be broadly similar albeit slight variations in the detailed experiences. The strength of our study was that we conducted interviews soon after participants had left quarantine to minimise recall bias after a change of environment. The study provides important information on quarantine experiences in institutions during COVID-19 and how to address them. Such information was missing in the literature at the time this study was conducted.

## Conclusion

Individuals in quarantine faced both negative and positive experiences influenced by the quarantine environment, quarantine management, their individual factors and linkage to other services. To improve the experience of quarantined individuals and reduce its associated negative impact, the pre-quarantine process should be better managed to comply with standards, quarantined persons should be provided as much information as possible, their quarantine duration should be kept short and costs of the process ought to be minimised. Furthermore, quarantine facilities should be assessed for suitability and monitored to comply with guidelines, avenues for access to healthcare for the quarantined need to be arranged and any potential stigma associated with quarantine thoroughly addressed.

## Supplementary Information

**Additional file 1.** In-depth interview guide.

**Additional file 2.** Completed COREQ checklist.

## Data Availability

The data/de-identified transcripts used during the current study are available from the corresponding author on reasonable request.

## Abbreviations

COVID-19: Corona Virus Disease 2019
SARS-CoV-2: severe acute respiratory syndrome coronavirus 2
