# Supplementary material for: Experiences of persons in COVID-19 institutional quarantine in Uganda: a qualitative study
Source: BMC Public Health. 2021 Mar 11;21:482. doi: 10.1186/s12889-021-10519-z (PMC7947936; doi:10.1186/s12889-021-10519-z)
Supplement: Supplementary file 1 — Additional file 1. In-depth interview guide. [file 12889_2021_10519_MOESM1_ESM.docx]

**Experiences of persons in COVID-19 institutional quarantine in Uganda: A qualitative study**

Rawlance Ndejjo, Gloria Naggayi, Ronald Tibiita, Richard Mugahi, Simon P.S. Kibira

**In-depth interview guide**

1. Under what circumstances did you find yourself in quarantine (institutional or self)?
2. How did you feel when you were told that you would stay in quarantine (institutional or self) for 14days?
3. What was your experience under quarantine (institutional or self)?

Probe for a) challenges, b) opportunities, c) coping mechanisms

1. What were your fears being in the quarantine? *(probes: risk of infection? Financial woes, other responsibilities, psychological distress etc.)*
   1. How about when leaving quarantine? Did you have any fears *(probe: stigma, financial issues)*
2. Was your quarantine duration extended? If so, why was it extended? How did you feel? What did this mean to you? How did you deal with it?
3. Were you aware about the quarantine (safety) measures in place? How did you know about them? Who communicated them? Were they clearly communicated to you?
   1. Probe for those not aware of any measures, explain how come they didn’t know any. Did you come up with your own measures? Please explain.
4. **If in institutional quarantine**: were you satisfied with how your institution followed the provided ministry of health COVID quarantine guidelines. Please tell me more about this.
   1. How can compliance with the guidelines be improved?
5. Overall, what should be done to improve the quarantine measures in self-quarantine?
6. Overall, what should be done to improve the quarantine measures in institutional quarantine?

*IF NOT MENTIONED IN THE INTERVIEW: Collect demographic data including age, sex, marital status, occupation and country of travel, do you have any dependents*

***Thank you for your time.***
